# Climate Change, Pesticides and Health: Considering the Risks and Opportunities of Adaptation for Zimbabwean Smallholder Cotton Growers

**DOI:** 10.3390/ijerph18010121

**Published:** 2020-12-26

**Authors:** Cliff Zinyemba, Emma Archer, Hanna-Andrea Rother

**Affiliations:** 1 Division of Environmental Health, and Centre for Environmental and Occupational Health Research, School of Public Health and Family Medicine, University of Cape Town, Observatory, Cape Town 7925, South Africa; cliff.zinyemba@gmail.com; 2 Department of Geography, Geoinformatics and Meteorology, University of Pretoria, Private Bag X20, Hatfield 0028, South Africa; emma.archer@up.ac.za

**Keywords:** health risks, incremental adaptation, maladaptation, transformational adaptation, pesticides, smallholder farmers, Zimbabwe

## Abstract

There is potential for increased pesticide-related adverse health outcomes in the agricultural sector linked to adaptive increases in pesticide use necessitated, in part, by climate change-related increases in pest populations. To understand the role of adaptation practices in pesticide use and health risks, this study assessed Zimbabwean smallholder cotton farmers’ adaptive responses linked to their climate change perceptions. In depth interviews were conducted with 50 farmers who had been growing cotton for at least 30 years. The study identified farmers’ adaptation practices that increased their pesticide use, as well as those that presented opportunities for reducing pesticide use through non-pesticide-dependent adaptation pathways. The findings show that due to perceived climate change impacts, such as a shorter growing season, farmers were adopting a range of adaptive practices. These included changes in pest management practices, such as increasing pesticide spraying frequencies due to keeping ratoon crops, which were increasing farmers’ overall pesticide use. Such incremental adaptive practices are potentially maladaptive, as they may increase farmers’ pesticide-related health risks. Other practices, however, such as reducing cotton acreage and diversifying crops, resulting in transformational adaptation, suggest the existence of opportunities for decreasing overall pesticide use or totally eliminating pesticides from the farming system.

## 1. Introduction

Pesticides are associated with a range of acute and chronic adverse human health effects that compromise health-related quality of life [1,2]. Acute effects of pesticide exposure, such as skin irritation, nausea, vomiting, headache, dizziness, and eye irritation, among other effects, are experienced immediately after exposure, and are often associated with singular short-term exposures [3,4]. Chronic effects are associated with long-term pesticide exposure and can manifest in a range of forms, including carcinogenic, endocrine disrupting, reproductive, developmental, neurological, immunotoxic and genotoxic adverse health effects [5,6,7,8]. A considerable body of research has demonstrated that varied factors, including political, economic, social and personal factors interact to jointly impact pesticide exposure and, thus, related adverse health outcomes [9,10]. In recent years, the role of climate change in pesticide health risks has, increasingly, been considered due to its potential for acting as an additional risk factor in pesticide exposure [11]. Evidence, for example, suggests that climate change-related increases in temperature may lead to pest population growth for certain species [12,13,14]. Warmer temperatures may, in addition, result in accelerated dissipation of pesticides by the processes of volatilisation and photodegradation [15,16,17]. To cope with these counteracting processes, farmers could resort to increasing the volume and frequency of pesticide applications as an adaptation mechanism. Adaptation can be defined as the act of taking measures to enhance capacity to cope with the effects of climate change by, for instance, adjusting or completely changing practices [18,19,20]. However, for farmers to make adaptation decisions, they may have to perceive climate change impacts on their farming systems. Several studies in different parts of the world have shown that there is a link between farmers’ perceptions and their adaptation actions to climate change [21,22,23,24].

In this article, we explored pesticide health-related risks and benefits of adaptation in the pesticide-intensive Zimbabwean smallholder cotton farming sector. In Zimbabwe, existing research shows that climate change is already altering the country’s natural farming regions and pest habitat ranges, with potential to impact pesticide use [25,26,27,28]. Study findings suggest that by the year 2080, the areas climatically suitable for growing cotton would have significantly increased in the country [29]. This could have adverse implications for farmers’ health as increases in the use of pesticides for adaptation may be expected. In light of these possible impacts, understanding the health risks associated with pesticide-dependent approaches and exploring the health benefits associated with alternative methods would be important for decision making.

## 2. Theoretical Framing

Decision making theory suggests that farmers tend to adapt to the effects of climate change by applying an incremental mode of decision making which enhances established coping solutions [30,31,32]. Thus, for farmers who use pesticides, increasing pesticide application may be considered a form of incremental adaptation [31,33]. Characteristically, incremental adaptation focuses on ensuring continuation of desired crop production systems as climatic and environmental contexts change [33]. An alternative perspective suggests that adaptation may be transformational, characterised by interventions that fundamentally change the various components of a crop production system [19,31,34,35]. Transformational adaptation by cotton farmers may take a range of forms, including crop switching and farm system transformation by, for instance, switching to animal husbandry. When adaptation results in additional risks affecting other systems than initially posed by climate change, it may be considered to be maladaptation [36,37]. In the case of pesticide-based adaptation, the increased use of pesticides may be maladaptive when the potential for the risk of pesticides exposure and resulting chronic and acute health effects increases.

## 3. Materials and Methods

### 3.1. Study Area

The research presented here, conducted from July to December 2015, was centred on Rushinga district, located in the northwestern part of Zimbabwe, where farmers use pesticides extensively for cotton production. Rushinga district covers part of the country’s natural farming region IV, which is hot, dry and sensitive to climate-related deviations in rainfall and temperatures. In the last population census in 2012, the district had a population of 74,000 and 17,000 households; while cotton production was the main source of income for approximately 90% of the households [38,39].

### 3.2. Data Collection

Ethical approval was granted by the Human Research Ethics Committee of the University of Cape Town’s Faculty of Health Sciences (HREC Ref: 300/2015). In Zimbabwe, further approval was granted by the Ministry of Health and Child Care’s Epidemiology and Disease Control Directorate, as well as the Ministry of Home Affairs.

Semi-structured in-depth interviews (Supplementary Materials) were conducted with 50 Rushinga cotton farmers. The farmers were recruited for the study by snowball sampling when interviewed farmers provided contact information of other farmers meeting the study criteria [40,41]. All participating farmers had consistently used pesticides for at least 30 years, a period long enough to make climate change inferences [42,43]. The selection process of participants was previously published [44]. Participants were asked about their perceptions regarding temperature and rainfall, as well as whether they had observed any changes in the past thirty years. The recorded perceptions were collated and validated against recent analyses of rainfall and temperature change records by Nyakudya and Stroosnijder [45] and the Ministry of Environment, Water and Climate [46]. Thereafter, farmers were asked about changes in their farming practices being implemented, as ways of coping with observed changes in rainfall and temperature. Following initial analysis of farmers’ responses, interviews to confirm and corroborate farmers’ observations and practices were conducted with key three informants who were agricultural extension workers in Rushinga district. To ensure validity and reliability, questions regarding specific events and those seeking exact numerical measures were avoided, since climatic events may be subject to recall bias, as they may be wrongly remembered or misinterpreted [47].

### 3.3. Data Analysis

Qualitative data analysis software, NVivo (versions 11 and 12), was used for the management and coding of all interview transcripts. To understand farmers’ perceptions regarding climate change and their adaptation strategies, different coding methods were applied in a four-stage process, starting with structural coding, followed by attribute coding, descriptive coding and, finally, magnitude coding [48]. Structural coding, a first cycle coding method for the initial categorisation of large amounts of textual data, was used to code entire interview transcripts for further in-depth analysis within categories [48,49]. Data were first coded into five literature-derived a priori perceptions data categories regarding key climate change metrics and observed effects, namely: temperature, rainfall, growing season, acreage and cropping patterns [25,26,29,47] (Table 1). These five were chosen as they are the most relevant for adaptation for the study farmers.

Thereafter, all five categories were analysed in more detail using descriptive coding, which is a topic coding technique that summarises a passage by assigning to it topic words or phrases [48]. Descriptive codes to capture detailed perceptions were developed based on participant’s statements, which depicted change over time, namely: changes in temperature characteristics, changes in rainfall characteristics, changes in growing season, changes in cotton acreage, and changes in other crops grown (Table 1).

All descriptively coded sections of the transcripts were subjected to further and more detailed analysis using magnitude coding, a technique which adds a statistical texture to qualitative data by describing intensity or frequency of a variable of interest [48]. Magnitude codes developed illustrated the 50 participants’ perceptions regarding changes that have happened over the past 30 years concerning rainfall, temperature, cotton growing season, cotton acreage and cropping patterns. For example, analysing farmers’ responses to the first question in Table 1 regarding temperature change, three coding techniques in the order of structural (broad), descriptive (narrow) and magnitude (specific) coding were used. Magnitude coding was used to categorise the responses in the 50 perception questionnaires to indicate either an increase or a decrease in overall temperature.

## 4. Results

### 4.1. Participants’ Demographics

The study participants’ ages ranged from 54 to 73 years. Thirty-six of the 50 interviews were conducted with male heads of households, who had indicated that it was they who had actively carried out pest management duties, such as pesticide spraying, on their farms in the past 30 years. Nine interviews were conducted with female heads of households, and the remainder (*n* = 5) were conducted with male–female couples, who indicated that they had both been equally involved in pest management activities.

### 4.2. Farmers’ Perceptions Regarding Climate Change

All participating farmers believed that the local climate had changed in some way. They identified effects in terms of three key climate change metrics of interest, namely: increase in the average atmospheric temperature (84%), average decline in total rainfall (54%) and shortening of the growing season (89%) (Table 2).

#### 4.2.1. Changing Temperature Patterns

Most of the study participants (84%) believed that temperatures in Rushinga had become warmer in the past 30 years. Fifteen percent believed there had been no change, while none mentioned it becoming cooler (Table 2). Almost three quarters of the participants who believed temperatures were increasing in Rushinga, indicated that summers were hotter, characterised by episodes of above average and extremely hot temperatures above 40 ⁰C. Participants further observed that winters were warmer, in comparison to the early 1980s. Several participant quotes illustrate these perceptions in changes in temperature over the past 30 years: “Since we have no means of measuring the temperatures, we cannot be very sure. However, I believe that the way it is hot nowadays is so different from how it was in the past” (Participant CZ 08). “Yes, temperatures have changed a lot. It’s now much hotter than in the past” (Participant TM 04). “There has been a change in temperature. It is now warmer than in the past. High temperatures used to be associated with the rains, but nowadays it just gets too hot without any rains falling” (Participant TM 26).

#### 4.2.2. Changing Rainfall Patterns

Fifty-four percent of respondents noted that the overall seasonal amount of rainfall had declined, while forty-four percent reported no changes in seasonal quantity (Table 2). Just one of the farmers reported an increase in seasonal rainfall amounts. There was some consensus in farmer perceptions that rainfall patterns reflected changes in the annual variability. Such respondents described rainfall as becoming more sporadic, and reported droughts as being more frequent. The number of rainy days per year were seen as reducing, as shown by the following comments: “Yes, there are great changes. We are no longer receiving any rainfall. During the rainfall season, we can count the number of days that it rains meaningfully, maybe just three times, the whole season. When the rain goes, it goes for good. Around March, we are no longer receiving any rains like we used to in the past” (Participant TM4). “I have only noticed that the way it rains now is different from how it rained in the past. In the past, by the 24th October we would have already received rainfall and planted our crops. In the recent years, however, we are looking at around Christmas time to start receiving our first rains” (Participant CZ4). “Yes, there is a big difference. In the past we would have rains till March. Nowadays the rains just come all at once, say starting around the beginning of December, then when it stops raining in February, that will be it; the end of the rain season” (Participant CZ17).

#### 4.2.3. Shorter Growing Season

Most participants (98%) indicated that the cotton growing season had become shorter, as compared to 30 years previously (Table 2). Only one participant was of the view that the length of the growing season had not changed, and none of the farmers believed the season had become longer. Participants observing a shorter season also described the growing season as shifting to starting later and ending early. Whereas in the past, the cotton growing season was six months long, commencing in October and ending in March, observations by farmers suggest that it has shifted and shortened—with onset in mid-December, ending towards the end of February or the beginning of March, as illustrated by the following comments: “Yes, the growing season has changed. In the past, farmers would have prepared their fields and put some lines in their fields and planted their cotton around the 15th of October, but these days people are getting way into December before they have prepared their fields” (Participant CZ1). “The season has changed because the rains are coming late, and they are leaving us early. So, the season is now very short. In the past, we had rains from around October till March or April” (Participant CZ4).

### 4.3. Adaptation Strategies

#### 4.3.1. Incremental Adaptive Changes in Pest Management Practices

As a way of adapting to the shorter season, participating farmers reported a new, but illegal, practice prevalent in the past 10 to 15 years, of keeping residue crop from the previous season, called ratoon cotton. Participants indicated that the changing season played a role in their reluctance to destroy cotton stalks as legally required to reduce bollworm breeding, as illustrated in the following quotes: “The season has changed. By now, I should have cut my cotton stalks and already prepared my land. But they are still standing in the field, and it’s October. The season is now starting very late. Its starting even on the 15th of December” (Participant CZ8). This was corroborated by one key informant who is an agricultural extension officer who said the following: “There has been a big change; pests have increased in their population… farmers are no longer cutting and burning their cotton stumps. Those farmers who do not cut and burn them end up maintaining their ratoon crops which are pests infested. By the time the rains come, bollworms and their eggs will already be in the plants” (KI 01). It was also noted that the frequency with which farmers sprayed their crops in a season had increased compared to when they started growing cotton in the early 1980s. Some farmers held the opinion that pesticides were no longer effective in controlling pests.

#### 4.3.2. Transformational Adaptive Changes in Farming Systems

Ninety-two percent of farmers reduced their average cotton acreage from 2.5 hectares during the 1980s and early 1990s, to just over one hectare at the time of the study, in part as a way of adapting to climate change (Table 2). These farmers cited low yields due to poor rains and increasing pest populations, as some of the main reasons responsible for the reduction in cotton acreage. Other reasons cited included a persistent low market price that had acted as a disincentive, high input costs and old age, as the following quotations illustrate: “We have reduced our cotton acreage and increased that of maize because maize production does not need intensive use of pesticides. We have also increased our groundnuts acreage because with ground nuts we can make peanut butter and sell” (Participant CZ 24). “I have considered that in future I should completely stop growing cotton and concentrate on the other crops. There have been major changes in harvests per acreage mainly because of the changes in weather conditions. The harvests that we used to have in the past when we used to receive reliable rainfall are so different from the harvests we are currently having per hectare” (Participant CZ 25). “Yes, there have been changes. In the past cotton was doing very well, but nowadays it is not growing well, and there are now a lot of pests, that is why I am just increasing the acreage of ground nuts and maize” (Participant TM 23). Only 4% of farmers reported having maintained their acreage, while another 4% increased their acreage by between half and one hectare, hoping to maintain the same level of cotton income in the context of falling yields and poor market prices. All 50 farmers reported that they had diversified cash crop types grown on their farms due to perceived mean annual rainfall variability, the changing growing season and persistent non-commensurate low cotton revenue. On average, two major crops were grown by participating farmers during the 1980s and the early 1990s—maize for subsistence and cotton as a cash crop. Participants reported having increased their average production to four crops, with the addition of some small grains for subsistence and ground nuts as a cash crop, starting from the late 1990s.

## 5. Discussion

Zimbabwean smallholder farmers of Rushinga district were asked about their perceptions regarding climate change and, thereafter, how they were adapting to the impacts of climate change on cotton farming and whether these measures implemented were increasing or reducing the use of, and health risks from pesticides. As already stated, perceptions regarding climate change serve as a support for implementing adaptation decisions and actions in smallholder agriculture [22,47,50,51]. Farmers’ reported climate change impacts correlated with the findings in other studies.

Farmers’ perception of warming temperatures was consistent with available climatological evidence for Southern Africa, which shows that the whole region has experienced an overall increase in temperature over the recent past [52,53]. Zimbabwe, in particular, has experienced a slightly higher rate of warming than the regional average, and is expected to continue with this trend, due to its continental interior location, which makes it prone to more rapid warming [46]. With regard to rainfall, participants’ perceptions seemed to concur with earlier analyses that suggested a decrease in rainfall [54,55,56,57,58]. However, more recent analyses indicate that climate change effects on rainfall are not yet statistically significant within the available historical rainfall record stretching back to 1920 [59]. Nyakudya and Stroosnijder (2011) analysed Rushinga district’s rainfall data for the period 1980–2009, and found that the district had not experienced a statistically significant decline in rainfall amount during that period. They observed high variability for both annual and seasonal rainfall totals, however, with high incidence of droughts, which agrees with farmers’ observations. While farmers perceived that the cropping season was shifting, currently there are limited published studies that show evidence of shifting growing seasons in Zimbabwe. A study on farmers’ climate change perceptions carried out in two Zimbabwean districts by Moyo and colleagues (2012) showed, however, that farmers largely believed that the rainy season had shifted—starting late, and ending early and abruptly. There are recent observations of late onset of rains over other places in Southern Africa [60], suggesting the possibility of a regional shift in the growing season.

The perceived changes in all three key climate change metrics of interest were reflected in farmers’ reasoning for implementing some adaptation strategies. In the study district of Rushinga, the indication is that perceptions of a shifting season might be triggering some incremental adaptive responses, shaping overall pesticide use in the district’s cotton production system. For instance, previously published results highlight that, from the early 1980s, cotton growers recorded increases in both pest populations and pesticide use due to, among other factors, farmers’ perceptions regarding climate variability and change [44]. To adapt to the shorter season, farmers increasingly found keeping ratoon cotton as an attractive strategy. The ratoon crop can be harvested in a shorter time than a newly planted one, as it is characterised by a well-established root system which enables it to survive long dry spells [61]. Farmers, therefore, found ratoon cotton to be more suited to a shorter and drier season. Ratoon cotton, however, provides shelter to pests, such as bollworms, against which farmers use regulated broad-spectrum pyrethroid pesticides, such as lambda-cyhalothrin, fenvalerate and deltamethrin, much earlier in the season than is gazetted [62]. For instance, in Rushinga, pyrethroids are supposed to be used between 25 December and 28 February only [62,63]. However, as bollworms harboured by the ratoons appear much earlier, farmers spray pyrethroids as early as the beginning of November—thereby compromising the opportunity for biological insect control [63]. Without biological control, farmers become increasingly dependent on pesticides to control pests. As a climate change adaptive practice, ratoon cropping could significantly increase the use of pyrethroid pesticides linked to health effects such as neurodevelopmental disorders, adverse behavioural problems in children after utero exposure, brain tumours, congenital abnormalities of the male reproductive system, adverse pregnancy outcomes, among others [64,65,66].

Intensified pesticide application for adaptation purposes may, as discussed earlier, temporarily control pest problems but end up being maladaptive—as this practice may unwittingly result in increased pesticide exposures and associated health risks [3,36,37,67]. A limitation of the present study is that data on pesticide exposure were not gathered and, therefore, conclusions based on the prevalence of pesticide poisoning in the study area cannot be made. However, several researchers have expressed concern over high levels of pesticide poisoning in the Zimbabwean smallholder farming sector [61,68,69,70]. Health risks of particular concern, particularly in the low-and middle-income countries (LMICs) where pesticide exposures are high, are those associated with highly hazardous pesticides, such as endocrine disrupting pesticides. These pesticides act by mimicking hormones, compromising the optimal function of the organs and systems regulated by affected hormones to result in a range of chronic adverse health effects [6,71,72,73]. Since adverse health outcomes associated with endocrine disrupting pesticides may take decades to appear, even among children of those originally exposed [74,75], any adaptive measures influencing pesticide use decisions, potentially affect pesticide-related health risks both in the short-term and the long-term. There is, thus, a clear and urgent need for the strengthening and support of alternative less toxic adaption options through government regulations, including banning of highly hazardous pesticides and continuous training of extension agents.

In Rushinga, besides farmers following an incremental adaptation pathway, there was also an inclination towards less toxic adaptation options—for example, reducing average cotton acreage and growing other crops. These transformational adaptation strategies have several socioeconomic benefits, such as improving food security, minimising risks associated with failure of one crop to reach maturity and increasing yield stability [22,76,77]. Health benefits include reduced pesticide-related health risks as crop diversification provides farmers an opportunity to grow less pesticide-dependent crops. Thus, contrary to the expectation that climate change would lead to increases in pesticide use, some transformational adaptive options appear to create alternative opportunities for reducing pesticide use. In many LMICs where pesticide-related adverse health outcomes are considered to be high [78], there may be an opportunity for pesticide exposure minimisation that can be integrated as part of climate change adaptation through transformational adaptation planning. Smallholder farmers who use pesticides should be encouraged, through agricultural extension services, to diversify their agricultural ventures to facilitate transformational adaptation. A move into nonfarm occupations, especially by younger cotton farmers, may be an important transformational adaptation strategy with potential to result in long-term health benefits. The health sector can also play an important role through health education and promotion activities that equip farmers with knowledge regarding the harms of adaptive increases in pesticide use and the health benefits of reducing pesticide exposures. Through education, awareness and relevant policies, smallholder farmers should be able to implement transformational adaptations that promote health benefits.

## 6. Conclusions

Perceptions regarding climate change may elicit adaptive responses by smallholder farmers, which could amplify and perpetuate the use of pesticides with long-term health risks. There are, however, opportunities for reducing pesticide use, including improved national policies, strategies and extension support services. These opportunities could assist farmers in transitioning from growing pesticide intensive crops to those which do not depend on pesticides, as well as using less-toxic alternatives. Transformational adaptation planning that promotes alternative crops can maximise health benefits of adaptation for farmers as well as support agricultural sector resilience, thus allowing farmers to realize multiple benefits. It is important that pest and pesticide management feature more prominently in relevant climate change adaptation strategies.

## Figures and Tables

**Table 1:** Coding variables used for analysing interview responses.

Questions	Structural Codes	Descriptive Codes	Magnitude Codes
In the past 30 years, have there been any changes in temperature?	Temperature	Changes in temperature characteristics	Increase
			No change
			Decrease
Have there been any changes in rainfall in the past 30 years?	Rainfall	Changes in rainfall characteristics	Increase
			No change
			Decrease
			Shorter
Have changes in temperature and/or rainfall affected your cotton growing season in any ways?	Growing season	Changes in growing season	Longer
			No change
			Shorter
Have there been any changes in your cotton acreage in the past 30 years	Acreage	Changes in cotton acreage	Increase
			No change
			Decrease
Have there been any changes in your cropping patterns in the past 30 years?	Cropping patterns	Changes in other crops grown	Increase
			No change
			Decrease

**Table 2:** Participant perceptions in relation to key climate change metrics of interest and adaptive responses (*n* = 50).

Structural Codes	Descriptive Codes	Magnitude Codes	Magnitude Responses n (%)
Temperature	Changes in temperature characteristics	Increase	42 (84%)
		No change	08 (16%)
		Decrease	00 (0%)
Rainfall	Changes in rainfall characteristics	Increase	01 (2%)
		No change	22 (44%)
		Decrease	27 (54%)
Growing season	Changes in growing season	Longer	00 (0%)
		No change	01 (2%)
		Shorter	49 (98%)

## Data Availability

The data presented in this study are contained within the article and Supplementary Materials.

## Supplementary Materials

The following are available online at https://www.mdpi.com/1660-4601/18/1/121/s1.

## References

[1] Muñoz-Quezada M.T., Lucero B.A., Iglesias V.P., Muñoz M.P., Cornejo C.A., Achu E., Baumert B., Hanchey A., Concha C., Brito A.M. (2016). Chronic exposure to organophosphate (OP) pesticides and neuropsychological functioning in farm workers: A review. Int. J. Occup. Environ. Health.

[2] Xin F., Susiarjo M., Bartolomei M.S. (2015). Multigenerational and transgenerational effects of endocrine disrupting chemicals: A role for altered epigenetic regulation?. Semin. Cell Dev. Biol..

[3] Gerage J.M., Meira A.P.G., da Silva M.V. (2017). Food and nutrition security: Pesticide residues in food. Nutrire.

[4] Lekei E.E., Ngowi A.V., London L. (2014). Farmers’ knowledge, practices and injuries associated with pesticide exposure in rural farming villages in Tanzania. BMC Public Health.

[5] Richardson J.R., Fitsanakis V., Westerink R.H.S., Kanthasamy A.G. (2019). Neurotoxicity of pesticides. Acta Neuropathol..

[6] Mnif W., Hassine A.I.H., Bouaziz A., Bartegi A., Thomas O., Roig B. (2011). Effect of endocrine disruptor pesticides: A review. Int. J. Environ. Res. Public Health.

[7] Del Pino J., Moyano-Cires P.V., Anadon M.J., Díaz M.J., Lobo M., Capo M.A., Frejo M.T. (2015). Molecular Mechanisms of Amitraz Mammalian Toxicity: A Comprehensive Review of Existing Data. Chem. Res. Toxicol..

[8] Sugeng A.J., Beamer P.I., Lutz E.A., Rosales C.B. (2013). Hazard-ranking of agricultural pesticides for chronic health effects in Yuma County, Arizona. Sci. Total Environ..

[9] Jallow M.F.A., Awadh D.G., Albaho M.S., Devi V.Y., Thomas B.M. (2017). Pesticide risk behaviors and factors influencing pesticide use among farmers in Kuwait. Sci. Total Environ..

[10] Galt R.E. (2010). Scaling Up Political Ecology: The Case of Illegal Pesticides on Fresh Vegetables Imported into the United States, 1996–2006. Ann. Assoc. Am. Geogr..

[11] Gatto M.P., Cabella R., Gherardi M. (2016). Climate change: The potential impact on occupational exposure to pesticides. Ann. Ist. Super. Sanita.

[12] Ziska L.H. (2014). Increasing minimum daily temperatures are associated with enhanced pesticide use in cultivated soybean along a latitudinal gradient in the mid-western United States. PLoS ONE.

[13] Ladányi M., Horváth L. (2010). A Review of the Potential Climate Change Impact of Insect Populations - General and Agricultural Aspects. Appl. Ecol. Environ. Res..

[14] Bebber D.P., Ramotowski M.A.T., Gurr S.J. (2013). Crop pests and pathogens move polewards in a warming world. Nat. Clim. Chang..

[15] Delcour I., Spanoghe P., Uyttendaele M. (2015). Literature review: Impact of climate change on pesticide use. Food Res. Int..

[16] Rhodes L.A., McCarl B.A. (2020). An analysis of climate impacts on herbicide, insecticide, and fungicide expenditures. Agronomy.

[17] Bustos N., Cruz-Alcalde A., Iriel A., Fernández Cirelli A., Sans C. (2019). Sunlight and UVC-254 irradiation induced photodegradation of organophosphorus pesticide dichlorvos in aqueous matrices. Sci. Total Environ..

[18] Grothmann T., Patt A. (2005). Adaptive capacity and human cognition: The process of individual adaptation to climate change. Glob. Environ. Chang..

[19] Smith K.R., Woodward A., Campbell-Lendrum D., Chadee D.D., Honda Y., Liu Q., Olwoch J.M., Revich B., Sauerborn R., Field C.B., Barros V.R., Dokken D.J., Mach K.J., Mastrandrea M., Bilir T.E., Chatterjee M., Ebi K.L., Estrada Y.O., Genova R.C. (2014). Human Health: Impacts, Adaptation, and Co-Benefits Coordinating Lead Authors: Contributing Authors. Climate Change 2014: Impacts, Adaptation, and Vulnerability. Part A: Global and Sectoral Aspects. Contribution of Working Group II to the Fifth Assessment Report of the Intergovernmental Panel on Climate Change.

[20] Smit B., Wandel J. (2006). Adaptation, adaptive capacity and vulnerability. Glob. Environ. Chang..

[21] Jørgensen S.L., Termansen M. (2016). Linking climate change perceptions to adaptation and mitigation action. Clim. Chang..

[22] Jiri O., Mafongoya P., Chivenge P. (2015). Smallholder Farmer Perceptions on Climate Change and Variability: A Predisposition for their Subsequent Adaptation Strategies. J. Earth Sci. Clim. Chang..

[23] Li S., Juh Asz-Horv Ath L., Harrison P.A., Aszl O. (2017). Pint Er, L.; Rounsevell, M.D.A. Relating farmer’s perceptions of climate change risk to adaptation behaviour in Hungary. J. Environ. Manag..

[24] Mitter H., Larcher M., Schönhart M., Stöttinger M., Schmid E. (2019). Exploring Farmers’ Climate Change Perceptions and Adaptation Intentions: Empirical Evidence from Austria. Environ. Manag..

[25] Mugandani R., Wuta M., Makarau A., Chipindu B. (2012). Re-classification of agro-ecological regions of zimbabwe in conformity with climate variability and change. Afr. Crop. Sci. J..

[26] Chikodzi D., Zinhiva H., Simba M.F., Murwendo T. (2013). Reclassification of Agro-Ecological Zones in Zimbabwe—The Rationale, Methods and Expected Benefits: The Case of Masvingo Province. J. Sustain. Dev. Afr..

[27] Chemura A., Kutywayo D., Chidoko P., Mahoya C. (2015). Bioclimatic modelling of current and projected climatic suitability of coffee (Coffea arabica) production in Zimbabwe. Reg. Environ. Chang..

[28] Kutywayo D., Chemura A., Kusena W., Chidoko P., Mahoya C. (2013). The Impact of Climate Change on the Potential Distribution of Agricultural Pests: The Case of the Coffee White Stem Borer (Monochamus leuconotus P.) in Zimbabwe. PLoS ONE.

[29] Brown D., Chanakira R.R., Chatiza K., Dhliwayo M., Dodman D., Masiiwa M. (2012). Climate Change Impacts, Vulnerability and Adaptation in Zimbabwe.

[30] Woods B.A., Nielsen H.Ø., Pedersen A.B., Kristofersson D. (2017). Farmers’ perceptions of climate change and their likely responses in Danish agriculture. Land Use Policy.

[31] Rippke U., Ramirez-Villegas J., Jarvis A., Vermeulen S.J., Parker L., Mer F., Diekkrüger B., Challinor A.J., Howden M. (2016). Timescales of transformational climate change adaptation in sub-Saharan African agriculture. Nat. Clim. Chang..

[32] Rickards L., Howden S.M. (2012). Transformational adaptation: Agriculture and climate change. Crop. Pasture Sci..

[33] Loginova J., Batterbury S.P.J. (2019). Incremental, transitional and transformational adaptation to climate change in resource extraction regions. Glob. Sustain..

[34] Kates R.W., Travis W.R., Wilbanks T.J. (2012). Transformational adaptation when incremental adaptations to climate change are insufficient. Proc. Natl. Acad. Sci. USA.

[35] Lonsdale K., Pringle P., Turner B. (2015). Transformational adaptation: What It Is, Why It Matters and What Is Needed.

[36] Barnett J., O’Neill S. (2010). Maladaptation. Glob. Environ. Chang..

[37] Magnan A.K., Schipper E.L.F., Burkett M., Bharwani S., Burton I., Eriksen S., Gemenne F., Schaar J., Ziervogel G. (2016). Addressing the risk of maladaptation to climate change. WIREs Clim Chang..

[38] Zimbabwe National Statistics Agency (2012). Zimbabwe Population Census: Provincial Report Mashonaland Central.

[39] Masuka G. (2012). Contests and struggle: Cotton farmers and COTTCO in Rushinga district, Zimbabwe, 1999–2006. Geoforum.

[40] Merriam S.B., Tisdell E.J. (2016). Qualitative Research: A Guide to Design and Implementation.

[41] Noy C. (2008). Sampling knowledge: The hermeneutics of snowball sampling in qualitative research. Int. J. Soc. Res. Methodol..

[42] Mach K.J., Planton S., von Stechow C. (2014). IPCC Annex II: Glossary. Climate Change 2014: Synthesis Report. Contribution of Working Groups I, II and III to the Fifth Assessment Report of the Intergovernmental Panel on Climate Change.

[43] Maddison D. (2007). The Perception of and Adaptation to Climate Change in Africa.

[44] Zinyemba C., Archer E., Rother H.-A. (2018). Climate variability, perceptions and political ecology: Factors influencing changes in pesticide use over 30 years by Zimbabwean smallholder cotton producers. PLoS ONE.

[45] Nyakudya I.W., Stroosnijder L. (2011). Water management options based on rainfall analysis for rainfed maize (Zea mays L.) production in Rushinga district, Zimbabwe. Agric. Water Manag..

[46] Government of Zimbabwe (2014). Zimbabwe’s National Climate Change Response Strategy.

[47] Niles M.T., Mueller N.D. (2016). Farmer perceptions of climate change: Associations with observed temperature and precipitation trends, irrigation, and climate beliefs. Glob. Environ. Chang..

[48] Saldana J. (2013). The Coding Manual for Qualitative Researchers.

[49] Ellis N.R., Albrecht G.A. (2017). Climate change threats to family farmers’ sense of place and mental wellbeing: A case study from the Western Australian Wheatbelt. Soc. Sci. Med..

[50] Moyo M., Mvumi B.M., Kunzekweguta M., Mazvimavi K., Craufurd P. (2012). Farmer perceptions on climate change and variability in semi-arid Zimbabwe in relation to climatology evidence. Afr. Crop. Sci. J..

[51] Rankoana S.A. (2018). Human perception of climate change. Weather.

[52] Tadross M., Davis C., Engelbrecht F., Joubert A., Archer van Garderen E., Davis C. (2011). Regional scenarios of future climate change over southern Africa. Climate Risk and Vulnerability: A Handbook for Southern Africa.

[53] Davis-Reddy C.L., Vincent K. (2017). Climate Risk and Vulnerability: A Handbook for Southern Africa.

[54] Unganai L.S. (1996). Historic and future climatic change in Zimbabwe. Clim. Res..

[55] Nicholson S.E. (2001). A Semi-Quantitative Regional Precipitation Data Set for Studying African Climates of the Nineteenth Century, Part 1. Overview of the Data Set. Clim. Chang..

[56] Hulme M., Doherty R., Ngara T., New M., Lister D. (2001). African climate change: 1900–2100. Clim. Res..

[57] Fauchereau N., Trzaska S., Rouault M., Richard Y. (2003). Rainfall Variability and Changes in Southern Africa during the 20th Century in the Global Warming Context. Nat. Hazards.

[58] Chamaillé-Jammes S., Fritz H., Murindagomo F. (2007). Detecting climate changes of concern in highly variable environments: Quantile regressions reveal that droughts worsen in Hwange National Park, Zimbabwe. J. Arid Environ..

[59] Mazvimavi D. (2010). Investigating changes over time of annual rainfall in Zimbabwe. Hydrol. Earth Syst. Sci..

[60] Archer E., Landman W., Tadross M., Malherbe J., Weepener H., Maluleke P., Marumbwa F. (2017). Understanding the evolution of the 2014–2016 summer rainfall seasons in southern Africa: Key lessons. Clim. Risk Manag..

[61] Mubvekeri W., Bare J., Makaka C., Jimu F. (2014). Assessing the diversity and intensity of pesticide use in communal area cotton production in Zimbabwe. J. Ecol. Nat. Environ..

[62] Tibugari H., Mandumbu R. (2017). Pest resistance management strategies: A mini review of the case of cotton (Gossypium hirsutum) in Zimbabwe. Zimbabwe J. Sci. Technol..

[63] Mapuranga R., Chapepa B., Mudada N. (2015). Strategies for integrated management of cotton bollworm complex in Zimbabwe: A review. Int. J. Agron. Agric. Res..

[64] Pan C., Wang Q., Liu Y.P., Xu L.F., Li Y.F., Hu J.X., Jiang M., Zhang J.P., Zhang M.R., Yu H.M. (2013). Anti-androgen effects of the pyrethroid pesticide cypermethrin on interactions of androgen receptor with corepressors. Toxicology.

[65] Furlong M.A., Barr D.B., Wolff M.S., Engel S.M. (2017). Prenatal exposure to pyrethroid pesticides and childhood behavior and executive functioning. Neurotoxicology.

[66] Rotem R.S., Chodick G., Davidovitch M., Hauser R., Coull B.A., Weisskopf M.G. (2018). Congenital Abnormalities of the Male Reproductive System and Risk of Autism Spectrum Disorders. Am. J. Epidemiol..

[67] Barnett J., O’Neill S.J. (2013). Minimising the risk of maladaptation. Climate Adaptation Futures.

[68] Tagwireyi D., Ball D., Nhachi C.F. (2006). Toxicoepidemiology in Zimbabwe: Pesticide poisoning admissions to major hospitals. Clin. Toxicol..

[69] Mugauzi R., Mabaera B., Rusakaniko S., Chimusoro A., Ndlovu N., Tshimanga M., Shambira G., Chadambuka A., Gombe N. (2011). Health Effects of Agrochemicals among farm workers in commercial farms of Kwekwe district, Zimbabwe. Pan Afr. Med. J..

[70] Maumbe B.M., Swinton S.M. (2003). Hidden health costs of pesticide use in Zimbabwe’s smallholder cotton growers. Soc. Sci. Med..

[71] Schug T.T., Janesick A., Blumberg B., Heindel J.J. (2011). Endocrine disrupting chemicals and disease susceptibility. J. Steroid Biochem. Mol. Biol..

[72] Mostafalou S., Abdollahi M. (2013). Pesticides and human chronic diseases: Evidences, mechanisms, and perspectives. Toxicol. Appl. Pharmacol..

[73] McKinlay R., Plant J.A., Bell J.N.B., Voulvoulis N. (2008). Endocrine disrupting pesticides: Implications for risk assessment. Environ. Int..

[74] Kabir E.R., Rahman M.S., Rahman I. (2015). A review on endocrine disruptors and their possible impacts on human health. Environ. Toxicol. Pharmacol..

[75] Bergman A., Heindel J.J., Kasten T., Kidd K.A., Jobling S., Neira M., Zoeller R.T., Becher G., Bjerregaard P., Bornman R. (2013). The impact of endocrine disruption: A consensus statement on the state of the science. Environ. Health Perspect..

[76] Altieri M.A., Nicholls C.I. (2017). The adaptation and mitigation potential of traditional agriculture in a changing climate. Clim. Chang..

[77] Belay A., Recha J.W., Woldeamanuel T., Morton J.F. (2017). Smallholder farmers’ adaptation to climate change and determinants of their adaptation decisions in the Central Rift Valley of Ethiopia. Agric. Food Secur..

[78] Jørs E., Neupane D., London L. (2018). Pesticide Poisonings in Low- and Middle-Income Countries. Environ. Health Insights.

