# Supplementary material for: Climate Change, Pesticides and Health: Considering the Risks and Opportunities of Adaptation for Zimbabwean Smallholder Cotton Growers
Source: Int J Environ Res Public Health. 2020 Dec 26;18(1):121. doi: 10.3390/ijerph18010121 (PMC7794869; doi:10.3390/ijerph18010121)
Supplement: Supplementary file 1 [file ijerph-18-00121-s001.pdf]

Supplementary Material: Interview transcript excerpts

Change in Temperature

<Internals\\Transcripts\\CZ 01> - § 2 references coded [1,14% Coverage]
These years its much warmer than it used to be in the past
Well, it's clear in the way it is hot. In the past we did not experience such heat.
<Internals\\Transcripts\\CZ 02> - § 1 reference coded [0,88% Coverage]
Yes, the temperatures have gone up. In the past it was not as hot as it is now.
<Internals\\Transcripts\\CZ 03> - § 1 reference coded [1,12% Coverage]
Yes, the weather is changing. In the past it was not as warm as it is now. This year, in particular, has been very warm.
<Internals\\Transcripts\\CZ 04> - § 1 reference coded [0,41% Coverage]
I haven't really noticed any changes in the temperatures.
<Internals\\Transcripts\\CZ 05> - § 1 reference coded [0,42% Coverage]
Yes, there are now changes. It's now much warmer.
<Internals\\Transcripts\\CZ 06> - § 1 reference coded [3,29% Coverage]
Uhmm, there has been a change. I think these days its no longer as hot as it used to be. Nowadays there is a wind breeze that seems to lower the temperatures a bit. In the past we could not comfortably walk barefooted because the ground would be burning. But, these days one can easily walk without wearing any shoes.
<Internals\\Transcripts\\CZ 07> - § 1 reference coded [0,52% Coverage]
The temperatures are the same.
<Internals\\Transcripts\\CZ 08> - § 1 reference coded [2,02% Coverage]
Since we have no means of measuring the temperatures, we cannot be very sure, But, i feel that the way it is hot nowadays is so different from how it was in the past
<Internals\\Transcripts\\CZ 09> - § 1 reference coded [1,75% Coverage]
The heat that i have noticed is mainly this year. This year has been much warmer than the other years. But, i have not seen any difference in terms of the rest of the other years.
<Internals\\Transcripts\\CZ 11> - § 2 references coded [0,87% Coverage]
Yes, there is a change. But, a small change.
Then, there is also the period that is not during the rainy season, there is a cold period that is coming during the time when we will be expecting to be the period when rains are made in the skies by the heat.
<Internals\\Transcripts\\CZ 13> - § 1 reference coded [0,99% Coverage]
The heat seems to be the same, if we take a look at it. However, when it comes to rainfall that is where there is a change.
<Internals\\Transcripts\\CZ 16> - § 1 reference coded [4,40% Coverage]
These recent years are much hotter. In the past we would experience very high temperatures that we used to call *barwe* around January. That period of heat came after a wet spell in December, and then the January heat allowed us to do weeding in the fields. Because of the heat, the grass would instantly dry once weeded. Once the rains returned the temperatures would then drop down a bit. These days it's now just very high temperatures throughout.
<Internals\\Transcripts\\CZ 18> - § 1 reference coded [7,29% Coverage]
These observations are not really accurate, because there is a year that it is quite cold, then another one when it gets warmer, and another one when it gets exceedingly hot. So, it's really variable, and i cannot really say there is a trend.
<Internals\\Transcripts\\CZ 20> - § 1 reference coded [1,27% Coverage]
I do not see any changes in temperatures. This area has always been a very hot area. Nothing has changed.
<Internals\\Transcripts\\CZ 22> - § 1 reference coded [1,01% Coverage]

These recent years have really been increasing in terms of heat.
<Internals\\Transcripts\\CZ 23> - § 1 reference coded [0,87% Coverage]
I have not observed any changes in temperatures. The temperatures are the same. Rushinga has always been a very hot place.
<Internals\\Transcripts\\CZ 24> - § 1 reference coded [1,29% Coverage]
The temperatures in Rushinga, if we look at the past three years, its now much warmer than in the past. Even our harvests, if it rains, say on the 5th of November, if you do not plant on the 6th or the 7th, and plant only around the 15th, you will not harvest anything, because of the way it is now hot.
<Internals\\Transcripts\\CZ 25> - § 1 reference coded [0,87% Coverage]
There has been an increase in temperatures. The heat is now too much.
<Internals\\Transcripts\\TM 01> - § 1 reference coded [1,88% Coverage]
Yes, there are changes. In the past, august would get really hot. But these recent years i have noticed that the heat comes only in October and then there are also periods of cool weather.
<Internals\\Transcripts\\TM 04> - § 1 reference coded [0,68% Coverage]
Yes, temperatures have changed a lot, It's now much hotter than in the past.
<Internals\\Transcripts\\TM 05> - § 1 reference coded [0,89% Coverage]
It's getting warmer. In the past it was not as warm as it is now.
<Internals\\Transcripts\\TM 06> - § 1 reference coded [0,65% Coverage]
Yes. The recent years are much warmer.
<Internals\\Transcripts\\TM 07> - § 1 reference coded [0,57% Coverage]
There is a big difference. It's now much warmer.
<Internals\\Transcripts\\TM 08> - § 1 reference coded [1,68% Coverage]
Yes, nowadays it’s much warmer that it used to be in the past. In the past it really wasn’t as hot as this.
<Internals\\Transcripts\\TM 09> - § 1 reference coded [1,20% Coverage]
Yes, these recent years it is much warmer. It has increased in terms of temperatures.
<Internals\\Transcripts\\TM 10> - § 2 references coded [1,58% Coverage]
There is a change. Nowadays it’s exceedingly hot.
It's really getting hot,
<Internals\\Transcripts\\TM 11> - § 1 reference coded [0,97% Coverage]
Yes, there is a difference. Nowadays its really much hotter.
<Internals\\Transcripts\\TM 12> - § 1 reference coded [0,83% Coverage]
I think there are changes, particularly this year it is exceedingly hot.
<Internals\\Transcripts\\TM 13> - § 1 reference coded [1,04% Coverage]
Yes, there are changes, its getting warmer, and this year has been exceptionally warm
<Internals\\Transcripts\\TM 14> - § 1 reference coded [0,96% Coverage]
Yes, there are changes. It’s now different from 30 years ago. The way it is hot is increasing.
<Internals\\Transcripts\\TM 15> - § 1 reference coded [0,29% Coverage]
The heat is now extreme
<Internals\\Transcripts\\TM 16> - § 1 reference coded [1,62% Coverage]
Reference 1 - 1,62% Coverage
Yes, there have been changes. Its getting hotter, particularly over the past 7 years, the temperatures have significantly become hotter. In the past it was hot, but not to the extent of the past 7 years
<Internals\\Transcripts\\TM 17> - § 1 reference coded [0,49% Coverage]
Yes. There is a big change. This year is actually worse.
<Internals\\Transcripts\\TM 19> - § 1 reference coded [3,82% Coverage]
Yes. Nowadays it's very warm. In the past when it became really hot, we would know that it would rain, but these days it's just hot, but there are no rains.
<Internals\\Transcripts\\TM 20> - § 1 reference coded [0,92% Coverage]
Yes. These recent years are much warmer than in the past.
<Internals\\Transcripts\\TM 21> - § 1 reference coded [1,48% Coverage]

There is too much heat. The ground is now too dry for plant roots to reach.
<Internals\\Transcripts\\TM 22> - § 1 reference coded [0,58% Coverage]
Yes, there have been changes. Its now much warmer.
<Internals\\Transcripts\\TM 23> - § 1 reference coded [3,34% Coverage]
Yes, there have been changes, it's now different. In the past, when it was so hot we expected it to rain. We no longer understand the implications of such heat because it is not bringing any rains.
<Internals\\Transcripts\\TM 24> - § 1 reference coded [0,75% Coverage]
These recent years are much warmer than in the past.
<Internals\\Transcripts\\TM 25> - § 2 references coded [4,37% Coverage]
Yes, there have been some changes. In the past it was hot, but not as hot as it is now
We knew that when temperatures rose it would eventually rain, but nowadays no matter how hot it gets, the rains do not just come. Our crops wilt and get dry while in the fields.
<Internals\\Transcripts\\TM 26> - § 1 reference coded [3,12% Coverage]
There has been a change in temperature. It is now warmer than in the past. High temperatures were associated with the rains. But nowadays it’s just too hot, but the rains are not coming.
<Internals\\Transcripts\\TM 27> - § 1 reference coded [1,56% Coverage]
Yes. There are changes. In the past it was equally warm, but we knew that if it got so hot it would rain.

Change in rainfall

<Internals\\Transcripts\\CZ 01> - § 3 references coded [3,61% Coverage]
In those past decades it used to rain a lot, and these days the rains are so sporadic.
Yes, the growing season has changed. In the past, farmers would have prepared their fields and put some lines in their fields and planted their cotton around the 15th of October, but these days people are getting way into October before they have prepared their fields
It is also evident in the way it rains these days. During those years we had so much rainfall.
<Internals\\Transcripts\\CZ 02> - § 1 reference coded [2,69% Coverage]
There is a very big difference. The difference is as far apart as from here to Bulawayo (600km away). In the past we received a lot of rainfall such that we enjoyed farming, unlike these days. It’s now so different. There is no rain anymore.
<Internals\\Transcripts\\CZ 03> - § 3 references coded [3,13% Coverage]
In the past it used to rain a lot. But, in the recent years, we do not receive any rains
Well, it’s now only raining here and there, quite sporadically.
Well, i could say when it comes it rains heavily. But when it goes it goes for a long time. Receiving the same amount would be alright if it is spread over several episodes of rainfall.
<Internals\\Transcripts\\CZ 04> - § 3 references coded [3,90% Coverage]
I have only noticed that the way it rains now is different from how it rained in the past. In the past, by the 24th October we would have already received rainfall and planted our crops. In the recent years, however, we are looking at around Christmas time to start receiving our first rains.
The total amount has also changed. Its lower. We receive the rains from around December till February. Then the rains disappear. The rains are now very much limited.
The season has changed because the rains are coming late, and they are leaving us early.
<Internals\\Transcripts\\CZ 05> - § 2 references coded [2,50% Coverage]
There is also now a big difference. It’s raining less and less these days.
When the rains come, they come and spend a week falling. Then when it goes, it goes for a month. When it returns again, it's only to rain just a bit and then that will be it. So, you will not be able to harvest anything.
<Internals\\Transcripts\\CZ 06> - § 2 references coded [4,80% Coverage]
And when it rains, it just rains here and there, twice or thrice and then it stops, and the season is ended.
Well, the rain falls a in huge amounts sometimes when it comes, but that water never gets the chance to percolate into the ground. For agricultural purposes it would be better to have the same

rainfall amount, but spread over the whole season because crops grow in stages; there are times when the water is needed, then times when only the sun is needed.
<Internals\\Transcripts\\CZ 07> - § 2 references coded [3,14% Coverage]
I notice that there have been changes. We would have rains even in March. Nowadays, things are different.
There are rain years and then those other years that have no rains at all.
<Internals\\Transcripts\\CZ 08> - § 1 reference coded [1,74% Coverage]
We are getting the rains for a very short period. It's not even two months. We get our rains in December, and in January we get the last rain.
<Internals\\Transcripts\\CZ 09> - § 4 references coded [3,78% Coverage]
The way it is raining these days is different, it's lower.
It's stopping much earlier. It's coming on time, but then it goes much early.
Only the last season has been different. It rained a little more than average. But, the only problem that i notice with the rain is that it rains well, but the finishing is the problematic one.
The problem is the rains that have changed their pattern.
<Internals\\Transcripts\\CZ 10> - § 1 reference coded [0,82% Coverage]
In the past, we knew that the rains would come early and go early; in some cases it would come late and go late as well. It used to happen.
<Internals\\Transcripts\\CZ 11> - § 2 references coded [1,49% Coverage]
Especially, during the rainy season, there is that dry spell that often comes, and you can easily notice that plants will be wilting and drying up, even though it will be during the rainy season.
The rains are coming ''one time''. It just rains, say if it starts in December, it just rains a lot or mls, and when it does so, it does not rain throughout the country. Even here in Rushinga, when it rains it does not cover the whole district
<Internals\\Transcripts\\CZ 12> - § 1 reference coded [1,01% Coverage]
If it still rained like it used to in the past, like, say the 25th of September when the very first rains came, we would know that we would plant our cotton in October and have time to plant maize and groundnuts in November. Now, we are racing against time and the rains.
<Internals\\Transcripts\\CZ 13> - § 1 reference coded [1,23% Coverage]
Yes. When we started growing cotton we used to receive a lot of rainfall. However, in the recent years we have started noticing that the rain is now less
<Internals\\Transcripts\\CZ 15> - § 2 references coded [1,34% Coverage]
It may be the same amount, raining a lot, but in just one month. It is not spread over the whole rain season.
The pattern that i am observing is that the rain is coming in large amount but in one month. It is not spreading. But when it rains, sometimes the crops may even be waterlogged.
<Internals\\Transcripts\\CZ 16> - § 1 reference coded [2,76% Coverage]
Yes, there is a big difference. In the past we would have rains till March. Nowadays the rains just come all at once, say starting around the beginning of December, then when it stops raining in February, that will be it; the end of the rain season. So this has affected our season.
<Internals\\Transcripts\\CZ 17> - § 5 references coded [5,19% Coverage]
Yes, there is a big change. In the past it would rain, and we would have good harvests.
The recent years have been characterised by inconsistencies. Those who plant early are the ones who have a chance of getting a good harvest.
The change is that when we plant our crops do not receive sufficient rains.
The rains go before the crops are ripe,
Yes, the season has been affected. In the past, there were the first rains which were called *bumharutsva*. These rains were not for planting crops. People had to wait from the second rains to start planting. Nowadays, if you do not utilise the first rains you will be left behind. We now plant with bumharutsva rains.
<Internals\\Transcripts\\CZ 18> - § 3 references coded [20,05% Coverage]

Yes, there is a change. In the past, rain used to come and it rained very well. We used to have the chance to plant and weed our crops. However, nowadays when it rains, it rains all at once. When it stops, that is it, there will not be another episode of rains again. So, if you have your maize crop, and then it experiences this sudden and long break in rainfall, it will not bear any cobs.
However, in the past few years, we have been receiving our first rains in January, then a short break in February and the just a few showers in March. That’s the difference that we see in Rainfall changes.
The amount is also different. We are receiving much less rainfall now.
<Internals\\Transcripts\\CZ 19> - § 1 reference coded [2,18% Coverage]
In the past, around this time, the 15th of October, we would have already planted our cotton, knowing that by the end of October the rains would have fallen
<Internals\\Transcripts\\CZ 20> - § 2 references coded [2,76% Coverage]
Then March there is no rain. The way it is raining is affect the growing season. The amount of rain received has not changed, but the timing has. We now have a short season. The rains are now coming late, and leaving us early.
The amount of rain received has not changed, but the timing has.
<Internals\\Transcripts\\CZ 21> - § 1 reference coded [1,18% Coverage]
Yes, the rainfall has become lower than in the past
<Internals\\Transcripts\\CZ 22> - § 2 references coded [3,50% Coverage]
Yes, the rains are coming and then stopping suddenly. Then the amount of rainfall has declined. We used to have rains from November to March, nowadays it’s only coming on the 25th of December, and it’s not reaching March.
Then the amount of rainfall has declined.
<Internals\\Transcripts\\CZ 23> - § 2 references coded [1,57% Coverage]
We may even receive our first rains in December. When such rains come, they will be all, so we make use of the. The rains are essentially coming late these days, and then they are stopping early.
Yes, the amount has fallen.
<Internals\\Transcripts\\CZ 24> - § 3 references coded [2,18% Coverage]
There were the rains that came in August. We called these gukurahundi, washing off all the chaff from your harvested crops. Then there was bumharutsva rains that fell following the intense heat of the month of October, like now. Thereafter would fall the rains marking the growing season, and we would start growing.
It’s now starting to rain maybe on the 5th of December.
Nowadays our rains are stopping in February. In March we will have to dig with a hole in order to harvest our ground nuts. So, it’s different now
<Internals\\Transcripts\\CZ 25> - § 2 references coded [2,63% Coverage]
It used to start raining around the mid of October, then it would continue raining November, December, January, February. Otherwise in March we would still receive some rains.
. There is now drought after drought.
<Internals\\Transcripts\\TM 01> - § 1 reference coded [1,78% Coverage]
Yes, there are changes. There is a great difference. We knew that around 25 October to the beginning of November it would rain. But, nowadays the rain only comes around Christmas.
<Internals\\Transcripts\\TM 02> - § 2 references coded [2,06% Coverage]
In the past the rains were reliable and enough
But, nowadays there isn't. Today is actually 25 November, and we still haven’t received the rains.
<Internals\\Transcripts\\TM 04> - § 1 reference coded [2,80% Coverage]
Yes, there are great changes. We are no longer receiving any rainfall. During the rainfall season, we can count the number of days that it rains meaningfully, maybe just three times, the whole season. When the rain goes, it goes for good. Around March, we are no longer receiving any rains like we used to in the past.
<Internals\\Transcripts\\TM 05> - § 2 references coded [1,92% Coverage]

Yes, the rainfall is now very low.
Reference 2 - 1,45% Coverage
Rain is really unreliable. We no longer have any significant rains in February. In March there is nothing.
<Internals\\Transcripts\\TM 06> - § 1 reference coded [2,88% Coverage]
Yes, there are significant changes in rainfall patterns. In the 1980s, we received our rains early, such that by mid November, like now, we would have already planted.
<Internals\\Transcripts\\TM 07> - § 2 references coded [2,20% Coverage]
In March the rain is no longer falling.
In the past it would rain and fill the rivers, and when that happens we would not suffer drinking water shortage for ourselves and our livestock.
<Internals\\Transcripts\\TM 08> - § 2 references coded [2,01% Coverage]
The rains are now coming late, only around December.
On top of that, the amount of the rainfall itself has dramatically fallen.
<Internals\\Transcripts\\TM 09> - § 2 references coded [1,00% Coverage]
Yes. It is not raining well.
The rains are coming late, and going early.
<Internals\\Transcripts\\TM 10> - § 3 references coded [4,01% Coverage]
Yes, there are. It's now really like gambling. There are years that we do not receive any rains, then those when we receive generous amounts and get good harvests.
Yes, there are. It's now really like gambling. There are years that we do not receive any rains, then those when we receive generous amounts and get good harvests.
it's now raining less.
<Internals\\Transcripts\\TM 11> - § 1 reference coded [1,47% Coverage]
Yes, in the past it used to rain. Now it's different from nowadays. We had plenty of rains.
<Internals\\Transcripts\\TM 12> - § 2 references coded [4,28% Coverage]
Yes, there are changes. These years it no longer rains well as it used to in the past. The rainfall is now less than it used to be.
The timing has also changed. In the past, around this time in November, we could be finishing planting, then by December our crops would have grown to a reasonable height. Now it's so different, and the growing season has also been affected.
<Internals\\Transcripts\\TM 13> - § 1 reference coded [0,32% Coverage]
The water is insufficient.
<Internals\\Transcripts\\TM 14> - § 2 references coded [2,13% Coverage]
There is now a difference in the amount of water.
In the past, we would have two types of rain that would always come first. We had bumharutsva, then gukurahundi. And after these two, it would now start to rain
<Internals\\Transcripts\\TM 15> - § 1 reference coded [2,35% Coverage]
In the past, the rains used to come around the 15th of October. But, these past few years, about the past 5 or 6 years, the period which we are now receiving the rains is now different.
<Internals\\Transcripts\\TM 17> - § 1 reference coded [0,22% Coverage]
The rains are coming late.
<Internals\\Transcripts\\TM 19> - § 1 reference coded [0,81% Coverage]
There are now changes in rainfall
<Internals\\Transcripts\\TM 20> - § 1 reference coded [2,86% Coverage]
Yes, there are changes. In the past, by this time of the year (November) there would already be crops in the field. But nowadays the rains are coming late and we also plant late.
<Internals\\Transcripts\\TM 21> - § 3 references coded [3,98% Coverage]
Yes, the ''weather'' has changed. The rains are no longer coming at the expected time
The rains are also much less than in the past
They are also no longer predictable, and the season has therefore changed.

<Internals\\Transcripts\\TM 22> - § 1 reference coded [4,53% Coverage]
In the past we used to plant on the 14th of November, and the rains would come on the 18th. Then the rains would disappear for the first two weeks of December, and then return and we would continue working. We used to receive a lot of rainfall, and it rained till May. Nowadays if it rains in January and February, and when it stops mid-February it goes and don't even think that it will rain again.
<Internals\\Transcripts\\TM 23> - § 2 references coded [2,92% Coverage]
Yes, there have been changes, and the way it rains now is different from the way it used to rain in the past. This is in terms of the amount and the timing of the rainfall.
Yes, there have been changes, and the way it rains now is different from the way it used to rain in the past. This is in terms of the amount and the timing of the rainfall.
<Internals\\Transcripts\\TM 24> - § 1 reference coded [1,20% Coverage]
In the past when we used to grow a lot of cotton we used to receive a lot of rainfall.
<Internals\\Transcripts\\TM 25> - § 1 reference coded [1,03% Coverage]
The rains are coming late, and then they are also going early.
<Internals\\Transcripts\\TM 26> - § 2 references coded [3,83% Coverage]
The season has changed. In the past the rains would come with the heat of the sun, but nowadays there is no rain accompanying the heat.
In the past i used to receive sufficient rains, now i only receive more than sufficient sun.
<Internals\\Transcripts\\TM 27> - § 2 references coded [3,51% Coverage]
The rains are now only starting to come in January, and these have affected our cropping season. In the past we would plant by the 25th of October, and then around the first week of November it would be raining.
The rains are coming late

Changing Season

<Internals\\Transcripts\\CZ 02> - § 1 reference coded [2,08% Coverage]
Oh yes, in the past we knew that by November we will have tilled our land. In fact, we planted our crops in November, and the rains would fall while the seeds were already in the ground.
<Internals\\Transcripts\\CZ 03> - § 1 reference coded [1,60% Coverage]
Yes, in the past we would plant in November. We would go for Christmas having tendered our maize. But, nowadays we receive the rains in December, in November its just luck.
<Internals\\Transcripts\\CZ 04> - § 1 reference coded [1,36% Coverage]
The season has changed because the rains are coming late, and they are leaving us early. So the season is now very short. But, in the past, we had rains from around October till March/April.
<Internals\\Transcripts\\CZ 05> - § 1 reference coded [1,78% Coverage]
And this has had the effect of shortening the growing season cotton because people are now planting their cotton in December, whereas in the past people planted in October. There has been a shift of two months.
<Internals\\Transcripts\\CZ 06> - § 1 reference coded [0,87% Coverage]
The season is now starting late. We are basically beginning our season in December.
<Internals\\Transcripts\\CZ 07> - § 1 reference coded [5,88% Coverage]
Yes, the season has changed. By now i should have cut my cotton stalks and already prepared my land. But they are still standing in the field, and it's October. The season is now starting very late. Its starting even on the 15th of December. In the past, by the 27th on November our cotton would have been planted, growing in the fields.
<Internals\\Transcripts\\CZ 08> - § 1 reference coded [3,27% Coverage]
In the past even though it was hot, we used to have good harvests, but in the recent years the heat is making the season short, we have a short rain season now, as from mid February there is no more rain. The rains are now fewer, and the temperatures are now higher.
<Internals\\Transcripts\\CZ 09> - § 2 references coded [2,53% Coverage]
But i see that the main problem is that the rain season is coming to an end early.

Reference 2 - 1,71% Coverage
The season in the past started in November, ending in March. But these days the rain is coming in December, and February the rain is gone, and it’s no longer getting to March.
<Internals\\Transcripts\\CZ 10> - § 1 reference coded [1,67% Coverage]
Well, i could say there haven't been any changes. I could say what has changed is us the people. For example, we stop taking care of our livestock while our crops are still yet to be harvested from the fields. That is what is making us lose our crops and deprive us of good harvests.
<Internals\\Transcripts\\CZ 11> - § 2 references coded [2,49% Coverage]
Yes. In the past it used to rain in such a way that we expected certain months to have a certain amount of rainfall. In the past it used to start raining end of October. When i started growing cotton it started to rain in October. You see? That is in the past. October had its amount of rainfall. Then November till February had variable amounts. Nowadays, rainfall is starting to rain in November, and this would continue till the end of January, and after that, there would be no more rainfall.
Yes, definitely. Most of the farmers used to plant in October, but now they have changed. Sometime we even reach Christmas before we have planted anything. If you celebrate Christmas after having planted anything, sometimes it is cotton.
<Internals\\Transcripts\\CZ 12> - § 2 references coded [2,22% Coverage]
Yes, there are shifts in the seasons. While growing up, we would eat green mealies in December, but now we are only eating them in March. Our first effective rainfall is expected between the 15t and the 30th November. There are now disadvantages to the cotton which we normally planted by the 1st of November.
There is no time. If it still rained like it used to in the past, like, say the 25th of September when the very first rains came, we would know that we would plant our cotton in October and have time to plant maize and groundnuts in November. Now, we are racing against time and the rains.
<Internals\\Transcripts\\CZ 13> - § 1 reference coded [0,39% Coverage]
It's now shorter, compared to years in the past.
<Internals\\Transcripts\\CZ 14> - § 1 reference coded [0,93% Coverage]
If a farmer does not plant early, they will not harvest anything because by February/March the rains would have gone.
<Internals\\Transcripts\\CZ 15> - § 1 reference coded [2,91% Coverage]
In the 1980s, we received our first rains around September or October, or soon after people have finished harvesting. In the past if you had visited us in October like you have done now, you would not have found us sitting aimlessly like this. We would have been busy in the field, telling you that we are now left with ten days for us to start a normal cotton season. By the 25th of October, our cotton should be in the ground. But, because of the changes in the season, remember i said cotton needs six months in the field, our season now starts halfway, up to December, Christmas before cotton is planted or being planted
<Internals\\Transcripts\\CZ 16> - § 1 reference coded [2,76% Coverage]
Yes, there is a big difference. In the past we would have rains till March. Nowadays the rains just come all at once, say starting around the beginning of December, then when it stops raining in February, that will be it; the end of the rain season. So this has affected our season.
<Internals\\Transcripts\\CZ 19> - § 1 reference coded [4,13% Coverage]
I think there is a change. In the past, the rains used to come early, and then they would also go late. Now, the recent years have shown some changes. We now only get meaningful rains to start planting in December, and the February, March, the rains are gone. There is now a change in the season
<Internals\\Transcripts\\CZ 20> - § 1 reference coded [2,58% Coverage]
In the past, till the 1990s, we used to receive our rains early. By the end of November most people would have planted. But, since the 2000s, the rains start to come in December and continue raining till February.
<Internals\\Transcripts\\CZ 21> - § 1 reference coded [4,58% Coverage]

It's now also coming late. We used to receive rains early, say November, but it is now coming late in December. It is also now just leaving early before the crops have reached their ripening stage.
<Internals\\Transcripts\\CZ 22> - § 1 reference coded [1,49% Coverage]
Yes, it’s been affected we only start growing towards the end of December when the rains come.
<Internals\\Transcripts\\CZ 24> - § 2 references coded [1,23% Coverage]
Nowadays our rains are stopping in February. In March we will have to dig with a hole in order to harvest our ground nuts. So, it’s different now.
Yes, the season has changed because we used to plant in November, but now we are planting only in December. So, the growing season has changed.
<Internals\\Transcripts\\CZ 25> - § 2 references coded [4,14% Coverage]
Yes, there are some changes. The season is now shorter, the rains are coming late and they are also going early.
We used to plant, when it started to start in Mid-October such crops as cotton and into November till planting, then December we would be weeding. Nowadays we are only starting to plant in December, late December actually
<Internals\\Transcripts\\TM 01> - § 1 reference coded [1,78% Coverage]
Yes, there are changes. There is a great difference. We knew that around 25 October to the beginning of November it would rain. But, nowadays the rain only comes around Christmas.
<Internals\\Transcripts\\TM 04> - § 2 references coded [2,37% Coverage]
That has also changed. The rains are now coming late, and then it just stops raining mid-season.
The season has been cut short/ The rains are coming late and then they go in March while we will still be in the middle of the season before most our crops can be harvested.
<Internals\\Transcripts\\TM 05> - § 1 reference coded [0,44% Coverage]
The season has become very short
<Internals\\Transcripts\\TM 06> - § 1 reference coded [0,52% Coverage]
The seasons have changed, yes.
<Internals\\Transcripts\\TM 07> - § 1 reference coded [1,45% Coverage]
There are changes. Like i said, there is no longer rainfall in March. Our season is now only three months. If lucky four.
<Internals\\Transcripts\\TM 08> - § 1 reference coded [1,84% Coverage]
Yes, indeed. Our season has changed. We now have a three month growing season. In the past it was five to six months.
<Internals\\Transcripts\\TM 09> - § 1 reference coded [0,54% Coverage]
I mean the season is becoming shorter.
<Internals\\Transcripts\\TM 12> - § 1 reference coded [3,81% Coverage]
Our crops are not reaching maturity. When it rains we plant, and the crops grow, then the rains disappear, and then the crops dry because of lack of moisture. By the time the rains return, some of the crops fail to recover. When we plant new crops, they will not reach maturity before the rains finally go for the end of the season.
<Internals\\Transcripts\\TM 13> - § 2 references coded [4,16% Coverage]
You see now that we have not even tilled our land; we have not yet planted? So, tell me even when we plant? In which month are we now? December right! I do not know whether we shall plant on the 25th, or twenty what, i do not know, or whether how we are going to harvest, i wonder how we are going to do that.
Yes. The seasons have changed.
<Internals\\Transcripts\\TM 14> - § 1 reference coded [0,33% Coverage]
Yes, the season has also changed.
<Internals\\Transcripts\\TM 15> - § 1 reference coded [1,34% Coverage]
It has changed, it’s now different. Nowadays we are receiving rains during just two months, then it goes.
<Internals\\Transcripts\\TM 16> - § 1 reference coded [3,12% Coverage]

our season is only starting in December, after Christmas. If it starts early it may be around the 10th or the 15th of December. But, usually, we now expect proper rains after Christmas. The rains are stopping quite early despite the fact that they are coming late. In the past it used to rain till April, but nowadays by the end of February the rains would have already stopped and gone.
<Internals\\Transcripts\\TM 17> - § 2 references coded [1,85% Coverage]
In the past, during the 80s, we would get to Christmas while our crops are flowering in the fields. Nowadays by Christmas our seeds would still be in the house.
Yes, the season has definitely changed. It’s now short.
<Internals\\Transcripts\\TM 19> - § 1 reference coded [0,71% Coverage]
the seasons for growing crops
<Internals\\Transcripts\\TM 20> - § 1 reference coded [2,15% Coverage]
Yes, the season has changed. We still haven't planted, but in the past we would already been admiring our plants in the fields by now.
<Internals\\Transcripts\\TM 21> - § 1 reference coded [0,64% Coverage]
the season has therefore changed.
<Internals\\Transcripts\\TM 22> - § 2 references coded [5,10% Coverage]
In the past we used to plant on the 14th of November, and the rains would come on the 18th. Then the rains would disappear for the first two weeks of December, and then return and we would continue working. We used to receive a lot of rainfall, and it rained till May. Nowadays if it rains in January and February, and when it stops mid-February it goes and don’t even think that it will rain again.
In agriculture the season has become much shorter.
<Internals\\Transcripts\\TM 23> - § 1 reference coded [0,90% Coverage]
the growing season for the cotton has been shortened.
<Internals\\Transcripts\\TM 25> - § 1 reference coded [0,48% Coverage]
Yes. The season is now short.
<Internals\\Transcripts\\TM 27> - § 1 reference coded [3,11% Coverage]
The rains are now only starting to come in January, and these have affected our cropping season. In the past we would plant by the 25th of October, and then around the first week of November it would be raining.

Crop switching and mixing

<Internals\\Transcripts\\CZ 01> - § 1 reference coded [0,35% Coverage]
Yes, I also grow maize, *mhunga* and *mashava.*
<Internals\\Transcripts\\CZ 02> - § 2 references coded [3,02% Coverage]
Yes, alongside cotton we also grow maize and groundnuts.
In the beginning we were growing a lot of cotton. We had so much faith in the crop. But now, due to the problems associated with the market price of cotton, we have decided to grow maize for food. It's much better.
<Internals\\Transcripts\\CZ 03> - § 1 reference coded [0,39% Coverage]
I also grow maize, ground nuts and *mashava*
<Internals\\Transcripts\\CZ 04> - § 1 reference coded [0,24% Coverage]
I also grow maize and groundnuts.
<Internals\\Transcripts\\CZ 05> - § 1 reference coded [0,53% Coverage]
We also grow ground nuts, maize, cow peas, *mashava*, and *mhunga*.
<Internals\\Transcripts\\CZ 06> - § 1 reference coded [0,59% Coverage]
I also grow beans, and maize, and sun flowers and mhunga.
<Internals\\Transcripts\\CZ 07> - § 1 reference coded [0,68% Coverage]
I also grow maize, *mashava*, and *mhunga*.
<Internals\\Transcripts\\CZ 08> - § 1 reference coded [0,40% Coverage]
I grow maize, mhunga and mashava.

<Internals\\Transcripts\\CZ 09> - § 1 reference coded [0,32% Coverage]
I also grow maize and ground nuts
<Internals\\Transcripts\\CZ 10> - § 2 references coded [1,34% Coverage]
We also grow maize for food. We can not only grow cotton while we have nowhere to get food, that is why the acreage of cotton sometimes tend to get reduced when we grow more maize for food.
We now grow maize, sorghum, and rapoko.
<Internals\\Transcripts\\CZ 11> - § 3 references coded [0,99% Coverage]
I also grow maize, *mhunga, mashava*, and sometimes i grow *mhunga* enough to supply the whole of ward 13. There was one season in which i harvested 60 bags of mhunga, and i gave to people in need in the area.
No, *mhunga* is millet. Mashava is sorghum.
Maize! Each year i harvest 2 and half tonnes
<Internals\\Transcripts\\CZ 13> - § 1 reference coded [0,53% Coverage]
I also grow maize, ground nuts and *mashava* also known as *mapfunde*.
<Internals\\Transcripts\\CZ 15> - § 1 reference coded [0,26% Coverage]
I also grow ground nuts, maize, sunflowers and mashava.
<Internals\\Transcripts\\CZ 16> - § 1 reference coded [0,53% Coverage]
I also grow groundnuts, maize, *mashava, and mapfunde.*
<Internals\\Transcripts\\CZ 19> - § 1 reference coded [0,99% Coverage]
We also grow maize, and ground nuts, and mhunga, mapfunde and cow peas.
<Internals\\Transcripts\\CZ 20> - § 1 reference coded [1,51% Coverage]
We also grow maize and ground nuts on a sizeable acreage, then on much smaller portions we also grow cow peas and round nuts.
<Internals\\Transcripts\\CZ 21> - § 1 reference coded [1,57% Coverage]
I grow maize. I also intend to grow groundnuts as my main cash crop.
<Internals\\Transcripts\\CZ 22> - § 1 reference coded [0,82% Coverage]
I also grow maize, groundnuts, *mapfunde, and mashava*
<Internals\\Transcripts\\CZ 23> - § 2 references coded [1,14% Coverage]
I also grow maize and ground nuts. The acreage of cotton is actually getting smaller.
Yes, in groundnuts we often encounter aphids. We spray them with a pesticide.
<Internals\\Transcripts\\CZ 24> - § 2 references coded [1,02% Coverage]
I also grow maize, ground nuts, *mhunga, and mapfunde*, *zviyo,* and cow peas, round nuts.
In the past years we used to grow these and we would even partake in traditional harvesting ceremonies to give thanks to our ancestors for the good harvests
<Internals\\Transcripts\\CZ 25> - § 1 reference coded [0,66% Coverage]
I also grow maize, sunflowers, *mashava* and groundnuts
<Internals\\Transcripts\\TM 01> - § 1 reference coded [0,49% Coverage]
I also grow ground nuts, and maize, and cow peas.
<Internals\\Transcripts\\TM 02> - § 2 references coded [1,83% Coverage]
For me it's been mainly cotton, them maize, ground nuts and mashava.
Mashava are the same as *mapfunde*, here we call them *mashava*.
<Internals\\Transcripts\\TM 04> - § 1 reference coded [0,75% Coverage]
I also cultivate maize, ground nuts, *mashava*, and sunflowers on a very small portion.
<Internals\\Transcripts\\TM 06> - § 1 reference coded [0,65% Coverage]
I also grow mashava, mhunga and maize.
<Internals\\Transcripts\\TM 07> - § 1 reference coded [1,09% Coverage]
I also grow maize, cow peas, mhunga, and mashava.
I have been growing these all the years.
<Internals\\Transcripts\\TM 08> - § 1 reference coded [1,79% Coverage]
I grow maize, ground nuts and mashava. These are the other crops that i have always been growing alongside cotton.

<Internals\\Transcripts\\TM 09> - § 1 reference coded [0,55% Coverage]
I also grow maize, mashava and mhunga.
<Internals\\Transcripts\\TM 10> - § 1 reference coded [1,02% Coverage]
I grow maize and mhunga. But, its mainly maize.
<Internals\\Transcripts\\TM 11> - § 1 reference coded [0,54% Coverage]
I also grow maize and ground nuts.
<Internals\\Transcripts\\TM 12> - § 1 reference coded [2,96% Coverage]
Yes, it's now different because we are now growing groundnuts, maize, mashava, mhunga, and everything, we are planting. Our acreage will not be very big, but we try to make sure that we diversify and grow a bit of everything so that we can harvest something.
<Internals\\Transcripts\\TM 13> - § 1 reference coded [0,39% Coverage]
Maize and mashava and groundnuts
<Internals\\Transcripts\\TM 14> - § 1 reference coded [0,73% Coverage]
Yes, i also grow groundnuts, sunflowers, maize and cow peas, and mhunga.
<Internals\\Transcripts\\TM 15> - § 1 reference coded [0,57% Coverage]
We also grow groundnuts and maize and mhunga.
<Internals\\Transcripts\\TM 16> - § 1 reference coded [0,41% Coverage]
I also grow maize, mhunga, mashava and ground nuts.
<Internals\\Transcripts\\TM 17> - § 1 reference coded [0,34% Coverage]
I also grow ground nuts, maize, mashava.
<Internals\\Transcripts\\TM 19> - § 1 reference coded [1,79% Coverage]
I also grow maize, ground nuts, mhunga mashava, round nuts, and cow peas.
<Internals\\Transcripts\\TM 20> - § 1 reference coded [0,74% Coverage]
I also grow maize, round nuts and ground nuts.
<Internals\\Transcripts\\TM 21> - § 1 reference coded [0,84% Coverage]
Maize, ground nuts, cow peas and round nuts
<Internals\\Transcripts\\TM 22> - § 1 reference coded [1,80% Coverage]
I also grow maize and groundnuts. I do not grow a lot over the past two years because my wife died, and this has affected my farming activities and production.
<Internals\\Transcripts\\TM 25> - § 1 reference coded [0,55% Coverage]
I also grow ground nuts and maize
<Internals\\Transcripts\\TM 26> - § 1 reference coded [0,50% Coverage]
I also grow maize and mashava.
<Internals\\Transcripts\\TM 27> - § 1 reference coded [0,66% Coverage]
I also grow maize, ground nuts and sunflowers

Change in cotton acreage

<Internals\\Transcripts\\CZ 01> - § 6 references coded [4,16% Coverage]
In the past we used to grow a lot of cotton. Well, i would say our harvest has dramatically been reduced.
In the past we used to grow a lot of cotton. Well, i would say our harvest has dramatically been reduced.
I believe it is because they are giving us pesticides which do not have power. I might be able to grow cotton on a very big piece of land spraying very well, but the problem is that the worms are not dying.
I believe it is because they are giving us pesticides which do not have power. I might be able to grow cotton on a very big piece of land spraying very well, but the problem is that the worms are not dying.
In the past we used to dedicate much of the land and resources to cotton. But, nowadays we are growing less cotton and expanding the acreage of the other crops, maize, mhunga, mashava and even ground nuts.

In the past we used to dedicate much of the land and resources to cotton. But, nowadays we are growing less cotton and expanding the acreage of the other crops, maize, mhunga, mashava and even ground nuts.
<Internals\\Transcripts\\CZ 03> - § 2 references coded [2,97% Coverage]
Yes, my cotton acreage has dwindled relative to the other crops. I am cutting down on cotton because there is no water. Water shortage is the main cause. Cotton must be planted early. If you do late planting you will lose all of it to livestock. The rains are no longer reliable. Whan it rains the rainfall is sporadic.
Yes, my cotton acreage has dwindled relative to the other crops. I am cutting down on cotton because there is no water. Water shortage is the main cause. Cotton must be planted early. If you do late planting you will lose all of it to livestock. The rains are no longer reliable. Whan it rains the rainfall is sporadic.
<Internals\\Transcripts\\CZ 04> - § 4 references coded [2,76% Coverage]
The land needed for cotton is always larger, if you are to realise a good harvest. You need at least 3hectares.
The land needed for cotton is always larger, if you are to realise a good harvest. You need at least 3hectares.
Well, in the past when we used to get enough fertilisers we used to realise as much as 6/7 bales per hectare. Nowadays, because we no longer receive enough fertilisers, our harvests have declined. We are now realising just enough to survive, just about 2/3 bales per hectare.
Well, in the past when we used to get enough fertilisers we used to realise as much as 6/7 bales per hectare. Nowadays, because we no longer receive enough fertilisers, our harvests have declined. We are now realising just enough to survive, just about 2/3 bales per hectare.
<Internals\\Transcripts\\CZ 06> - § 2 references coded [3,07% Coverage]
Yes, the cotton acreage has declined mainly because of the crop's market price. I really loved and enjoyed my cotton, but i am now finding a crop that is selling for a better price on the market. A crop such as beans. I am planning to grow more beans and i have an area where i want to grow them.
Yes, the cotton acreage has declined mainly because of the crop's market price. I really loved and enjoyed my cotton, but i am now finding a crop that is selling for a better price on the market. A crop such as beans. I am planning to grow more beans and i have an area where i want to grow them.
<Internals\\Transcripts\\CZ 07> - § 2 references coded [0,73% Coverage]
Well, in my case there are always changes.
Well, in my case there are always changes.
<Internals\\Transcripts\\CZ 08> - § 2 references coded [4,22% Coverage]
Yes. The land used for cotton is getting smaller, because when we sell our cotton we are not getting good satisfactory returns. In the past if i sold a bale of cotton i would buy a cow, but now i cannot buy even a bar of soap. I they say i should get 50or 60 dollars for the bale, it means i am getting absolutely nothing for my crop and labour.
Yes. The land used for cotton is getting smaller, because when we sell our cotton we are not getting good satisfactory returns. In the past if i sold a bale of cotton i would buy a cow, but now i cannot buy even a bar of soap. I they say i should get 50or 60 dollars for the bale, it means i am getting absolutely nothing for my crop and labour.
<Internals\\Transcripts\\CZ 09> - § 3 references coded [9,94% Coverage]
The acreage of cotton is becoming smaller relative the other crops? It’s getting smaller because these days for the farmer to buy inputs, let me give an example, in the past when we used to get financial loans; if the loaners say 4 hectares of cotton, they would give you 8 fifty kg bags of D fertilisers, and 4 bags of top fertilisers. That time the prices were very affordable. Nowadays, you just receive just one or two bags. So, it’s now different, and people are now growing on small portions that are in line with the amount of inputs they have.

In the past through the 1980s we were growing cotton on a larger scale. But of late things have changed. This is also due to the rainfall which has also changed. The way it is raining these days is different, it’s lower. It’s stopping much earlier. It’s coming on time, but then it goes much early. That’s the problem which i see. Maybe the climate has changed, i wouldn’t know. But i see that the main problem is that the rain season is coming to an end early.
It’s getting smaller because these days for the farmer to buy inputs, let me give an example, in the past when we used to get financial loans; if the loaners say 4 hectares of cotton, they would give you 8 fifty kg bags of D fertilisers, and 4 bags of top fertilisers. That time the prices were very affordable. Nowadays, you just receive just one or two bags. So, it’s now different, and people are now growing on small portions that are in line with the amount of inputs they have.
In the past through the 1980s we were growing cotton on a larger scale. But of late things have changed. This is also due to the rainfall which has also changed. The way it is raining these days is different, it’s lower. It’s stopping much earlier. It’s coming on time, but then it goes much early. That’s the problem which i see. Maybe the climate has changed, i wouldn’t know. But i see that the main problem is that the rain season is coming to an end early.
<Internals\\Transcripts\\CZ 10> - § 2 references coded [1,08% Coverage]
We scaled down on cotton because of the market price; cotton is a very labour intensive crop and the market price has been going down. So we see it better to concentrate on food crops.
We scaled down on cotton because of the market price; cotton is a very labour intensive crop and the market price has been going down. So we see it better to concentrate on food crops.
<Internals\\Transcripts\\CZ 11> - § 2 references coded [1,38% Coverage]
Well, according with the tonnage that i harvest in cotton or maize, if i realise that it's better to increase my cotton acreage because i have a certain target, i just increase the cotton acreage. But the maize acreage remains the same because at times we harvest a lot of maize, much more than we can consume such that when we harvest we will still have plenty of maize from the previous season's harvest.
Well, according with the tonnage that i harvest in cotton or maize, if i realise that it's better to increase my cotton acreage because i have a certain target, i just increase the cotton acreage. But the maize acreage remains the same because at times we harvest a lot of maize, much more than we can consume such that when we harvest we will still have plenty of maize from the previous season's harvest.
<Internals\\Transcripts\\CZ 12> - § 4 references coded [3,39% Coverage]
My cotton acreage sometimes goes up, so i often rent land from others and grow up to 5 hectares of cotton in some seasons. I usually exceed 2 hectares when i have enough money to buy inputs. But, the other reason why i end up renting land from others is that i often do demo plots, usually with 10 to 12 different varieties. So, i then take out of these varieties and demonstrate to people during field days which varieties are most suited for our area of Rushinga. Then we explain to fellow farmers the advantages and disadvantages of different varieties. This is in the same line with the variety which i talked about earlier, the FQ904, which has fewer leaves.
My cotton acreage sometimes goes up, so i often rent land from others and grow up to 5 hectares of cotton in some seasons. I usually exceed 2 hectares when i have enough money to buy inputs. But, the other reason why i end up renting land from others is that i often do demo plots, usually with 10 to 12 different varieties. So, i then take out of these varieties and demonstrate to people during field days which varieties are most suited for our area of Rushinga. Then we explain to fellow farmers the advantages and disadvantages of different varieties. This is in the same line with the variety which i talked about earlier, the FQ904, which has fewer leaves.
No. They actually develop good big balls. In addition, the quality of the cotton is good such that it fetches a higher price on the market per kilo.
Yes, it is a bit expensive. They differentiate the pricing. I even grew once seed cotton as foundation.
<Internals\\Transcripts\\CZ 13> - § 2 references coded [0,57% Coverage]
Since i started my acreage was high, until recently when i down-scaled.

Since i started my acreage was high, until recently when i down-scaled.
<Internals\\Transcripts\\CZ 14> - § 4 references coded [2,98% Coverage]
People are no longer growing cotton as they used to. People have resorted to growing ground nuts and other small grains.
People are no longer growing cotton as they used to. People have resorted to growing ground nuts and other small grains.
i now prefer to grow more ground nuts. Early cropping. If you grow ground nuts early, you can harvest something meaningful and be able to send children to school.
When i was still working at Cottco, people used to get good returns from their cotton.
i now prefer to grow more ground nuts. Early cropping. If you grow ground nuts early, you can harvest something meaningful and be able to send children to school.
<Internals\\Transcripts\\CZ 15> - § 2 references coded [2,49% Coverage]
In the past our cotton acreage was largest. Not only ours; but everyone's. People would rather go without food crops after having dedicated the largest portion of land to cotton. It's because then, cotton was well paying. But, in the recent years the returns from cotton have been declining, and more and more farmers are now downscaling their cotton production. Some farmers are stopping completely growing cotton. The acreage of cotton relative to the other crops has drastically declined not only for me, but in the whole village.
In the past our cotton acreage was largest. Not only ours; but everyone's. People would rather go without food crops after having dedicated the largest portion of land to cotton. It's because then, cotton was well paying. But, in the recent years the returns from cotton have been declining, and more and more farmers are now downscaling their cotton production. Some farmers are stopping completely growing cotton. The acreage of cotton relative to the other crops has drastically declined not only for me, but in the whole village.
<Internals\\Transcripts\\CZ 19> - § 2 references coded [1,16% Coverage]
Well, the acreage always varies each year depending on how much we desire to grow.
Well, the acreage always varies each year depending on how much we desire to grow.
<Internals\\Transcripts\\CZ 20> - § 2 references coded [4,38% Coverage]
Yes, it has changed a lot. But, its mainly due to the issue of fertilisers. Our soils are spent, and for them to be productive we must apply fertilisers. The cotton companies, however, do not give much in terms of fertilisers when we get credit. Therefore because we also cannot afford to buy a lot of fertilisers, we have resorted to cutting the cotton acreage.
Yes, it has changed a lot. But, its mainly due to the issue of fertilisers. Our soils are spent, and for them to be productive we must apply fertilisers. The cotton companies, however, do not give much in terms of fertilisers when we get credit. Therefore because we also cannot afford to buy a lot of fertilisers, we have resorted to cutting the cotton acreage.
<Internals\\Transcripts\\CZ 22> - § 2 references coded [2,89% Coverage]
We have drastically cut our cotton acreage because of poor market prices. We have resorted to growing more maize. With maize, if you have fertilisers you can get a very good harvest.
We have drastically cut our cotton acreage because of poor market prices. We have resorted to growing more maize. With maize, if you have fertilisers you can get a very good harvest.
<Internals\\Transcripts\\CZ 23> - § 4 references coded [0,92% Coverage]
I also grow maize and ground nuts. The acreage of cotton is actually getting smaller.
I also grow maize and ground nuts. The acreage of cotton is actually getting smaller.
It's mainly because of the poor market price.
It's mainly because of the poor market price.
<Internals\\Transcripts\\CZ 24> - § 2 references coded [1,18% Coverage]
We have reduced our cotton acreage and increased that of maize because maize can be treated with pesticides and stored and kept for consumption into the next year. I have also increased my groundnuts acreage because with ground nuts i can cook and eat, i can make peanut butter.

We have reduced our cotton acreage and increased that of maize because maize can be treated with pesticides and stored and kept for consumption into the next year. I have also increased my groundnuts acreage because with ground nuts i can cook and eat, i can make peanut butter.
<Internals\\Transcripts\\CZ 25> - § 2 references coded [4,61% Coverage]
Yes, i have actually decided that i should completely stop growing cotton, and concentrate on the other crops. There have also been major changes in harvest per acreage mainly because of the changes in weather conditions. The harvests that we used to have in the past when we had more rains are now so different from the harvests that we are currently having per hectare.
Yes, i have actually decided that i should completely stop growing cotton, and concentrate on the other crops. There have also been major changes in harvest per acreage mainly because of the changes in weather conditions. The harvests that we used to have in the past when we had more rains are now so different from the harvests that we are currently having per hectare.
<Internals\\Transcripts\\TM 01> - § 2 references coded [3,31% Coverage]
Let me say that five years and back, cotton was of so much significance to me. My household was supported by cotton. Even my family survived on cotton. I bought my livestock using income earned from cotton. But, in the recent years the fact that the pesticides have become so weak, i cannot count on cotton any longer for survival.
Let me say that five years and back, cotton was of so much significance to me. My household was supported by cotton. Even my family survived on cotton. I bought my livestock using income earned from cotton. But, in the recent years the fact that the pesticides have become so weak, i cannot count on cotton any longer for survival.
<Internals\\Transcripts\\TM 04> - § 6 references coded [4,28% Coverage]
Yes, there have been changes. From 1980 to 2004, I have been doing very well, financially as cotton has been giving me good returns. But the post 2004 period has been very bad for me. I have, oftentimes, sold some of my live stock in order to have money.
Yes, there have been changes. From 1980 to 2004, I have been doing very well, financially as cotton has been giving me good returns. But the post 2004 period has been very bad for me. I have, oftentimes, sold some of my live stock in order to have money.
My family is living very well. I am actually now considering stopping with cotton so that I can just grow food crops.
My family is living very well. I am actually now considering stopping with cotton so that I can just grow food crops.
No, I still do the same acreage, but I am bitter that when I go to the market, cotton is fetching a very low price.
No, I still do the same acreage, but I am bitter that when I go to the market, cotton is fetching a very low price.
<Internals\\Transcripts\\TM 06> - § 2 references coded [5,79% Coverage]
Yes, the acreage of cotton is shrinking due to the shortage of inputs. With other crops the amount of inputs needed are not as many as those needed for cotton. In addition, with other crops, the stalks are useful, they can be tilled and become fertiliser to the soil, unlike cotton stalks which cannot be recycled to fertilise the soil.
Yes, the acreage of cotton is shrinking due to the shortage of inputs. With other crops the amount of inputs needed are not as many as those needed for cotton. In addition, with other crops, the stalks are useful, they can be tilled and become fertiliser to the soil, unlike cotton stalks which cannot be recycled to fertilise the soil.
<Internals\\Transcripts\\TM 07> - § 2 references coded [1,33% Coverage]
I grow maize and cotton on the largest portions. Then on smaller portions i grow cow peas, mashava, and mhunga.
I grow maize and cotton on the largest portions. Then on smaller portions i grow cow peas, mashava, and mhunga.
<Internals\\Transcripts\\TM 08> - § 4 references coded [5,95% Coverage]

Right now it’s not easy to tell whether it is more or less because we have been scaling down on cotton production because of low prices.
Right now it’s not easy to tell whether it is more or less because we have been scaling down on cotton production because of low prices.
There have been changes in the level of farming because all my children are gone. I am now alone and i am also now old. So i have had to cut down on production. If i do not have any herbicides i only grow where i can manage to weed manually.
There have been changes in the level of farming because all my children are gone. I am now alone and i am also now old. So i have had to cut down on production. If i do not have any herbicides i only grow where i can manage to weed manually.
<Internals\\Transcripts\\TM 09> - § 6 references coded [4,49% Coverage]
I am just persevering on as cotton is a crop that i have experience in growing.
I am just persevering on as cotton is a crop that i have experience in growing.
The acreage of cotton is declining.
The acreage of cotton is declining.
The reason is mainly economical. In addition, the labour that we put in the production of cotton is not commensurate with the revenue that we get, so that has led to the scaling down of cotton production.
The reason is mainly economical. In addition, the labour that we put in the production of cotton is not commensurate with the revenue that we get, so that has led to the scaling down of cotton production.
<Internals\\Transcripts\\TM 11> - § 4 references coded [3,45% Coverage]
I am now growing more groundnuts, and i have cut down the cotton acreage.
I am now growing more groundnuts, and i have cut down the cotton acreage.
At my age, i cannot continue growing cotton because it i s labour intensive. I now find it very difficult for me to carry the knapsack sprayer.
At my age, i cannot continue growing cotton because it i s labour intensive. I now find it very difficult for me to carry the knapsack sprayer.
<Internals\\Transcripts\\TM 12> - § 4 references coded [5,51% Coverage]
Nowadays i am increasing my groundnuts acreage. In the past i did not use to grow a large acreage of groundnuts, but we used to grow more cotton. But i have realised that these days it’s now better to grow more groundnuts.
Nowadays i am increasing my groundnuts acreage. In the past i did not use to grow a large acreage of groundnuts, but we used to grow more cotton. But i have realised that these days it’s now better to grow more groundnuts.
Yes, it’s now different because we are now growing groundnuts, maize, mashava, mhunga, and everything, we are planting. Our acreage will not be very big, but we try to make sure that we diversify and grow a bit of everything so that we can harvest something.
Yes, it’s now different because we are now growing groundnuts, maize, mashava, mhunga, and everything, we are planting. Our acreage will not be very big, but we try to make sure that we diversify and grow a bit of everything so that we can harvest something.
<Internals\\Transcripts\\TM 13> - § 2 references coded [3,72% Coverage]
We are now prioritising food crops over cotton. Ground nuts are now giving better returns (compared to cotton, as commercial crops). This season we grew cotton and harvested 3 bales, but we did not realise any income. Cotton is not paying any more. We are dying for nothing. Cotton has a lot of labour.
We are now prioritising food crops over cotton. Ground nuts are now giving better returns (compared to cotton, as commercial crops). This season we grew cotton and harvested 3 bales, but we did not realise any income. Cotton is not paying any more. We are dying for nothing. Cotton has a lot of labour.
<Internals\\Transcripts\\TM 14> - § 4 references coded [3,83% Coverage]

It has changed. In the past we didn’t grow many crops. We mainly grew cotton, and we would grow cotton on the best soils during that time when it gave us good financial returns.
It has changed. In the past we didn’t grow many crops. We mainly grew cotton, and we would grow cotton on the best soils during that time when it gave us good financial returns.
Now, we are now growing more crops depending on the system of the rainfall. We also now have to reserve a portion for mashava, and mhunga and maize which means the cotton acreage is no longer that big.
Now, we are now growing more crops depending on the system of the rainfall. We also now have to reserve a portion for mashava, and mhunga and maize which means the cotton acreage is no longer that big.
<Internals\\Transcripts\\TM 15> - § 4 references coded [7,48% Coverage]
There is a slight change because the cotton acreage is slightly larger. The problem that we face is in our inability to weed all the crops in time and have good harvests. If we put most of our efforts in cotton weeding, we also have better cotton harvests. Maize is good in that we will have food at home.
No, it’s no longer the same acreage, there is a change.
How is that so?
Because some of my children are no longer staying with me at home, they are grown-ups and they have left. So, with the children that i now have at home my acreage is now smaller, which they can manage to work.
No, it’s no longer the same acreage, there is a change.
Because some of my children are no longer staying with me at home, they are grown-ups and they have left. So, with the children that i now have at home my acreage is now smaller, which they can manage to work.
<Internals\\Transcripts\\TM 16> - § 2 references coded [2,31% Coverage]
Well, that varies from season to season. There are some season when i place more value on maize and dedicate more land to it. I prefer to dedicate more land to maize and mhunga because these are food crops that give guarantee as far as food security is concerned when i get good harvests.
Well, that varies from season to season. There are some season when i place more value on maize and dedicate more land to it. I prefer to dedicate more land to maize and mhunga because these are food crops that give guarantee as far as food security is concerned when i get good harvests.
<Internals\\Transcripts\\TM 17> - § 4 references coded [2,47% Coverage]
Yes, i have cut down on my cotton acreage and increased the maize acreage.
Yes, i have cut down on my cotton acreage and increased the maize acreage.
Because i realised that cotton is no longer fetching a favourable price on the market. In addition, it is very difficult to manage a large acreage of cotton because it is both a pesticide and labour intensive crop.
Because i realised that cotton is no longer fetching a favourable price on the market. In addition, it is very difficult to manage a large acreage of cotton because it is both a pesticide and labour intensive crop.
<Internals\\Transcripts\\TM 19> - § 2 references coded [5,68% Coverage]
In the past my cotton acreage was smaller. Now i grow a larger acreage. I used to also work in the past, so i had a second source of income. Now that i don’t have another job, my cotton acreage has increased than it was in the past.
In the past my cotton acreage was smaller. Now i grow a larger acreage. I used to also work in the past, so i had a second source of income. Now that i don’t have another job, my cotton acreage has increased than it was in the past.
<Internals\\Transcripts\\TM 20> - § 2 references coded [3,65% Coverage]
I normally make my cotton size bigger because it is from cotton that I get my money. The maize acreage is slightly smaller. My groundnuts is also larger because that is my other cash crop from which i get money for school fess.

I normally make my cotton size bigger because it is from cotton that I get my money. The maize acreage is slightly smaller. My groundnuts is also larger because that is my other cash crop from which i get money for school fess.

<Internals\\Transcripts\\TM 21> - § 2 references coded [1,72% Coverage]

Yes. The acreage of cotton has declined while that of maize and groundnuts has increased

Yes. The acreage of cotton has declined while that of maize and groundnuts has increased

<Internals\\Transcripts\\TM 22> - § 2 references coded [1,03% Coverage]

Yes. I now grow cotton on a very small scale, and i have given part of my land to my sons.

Yes. I now grow cotton on a very small scale, and i have given part of my land to my sons.

<Internals\\Transcripts\\TM 23> - § 2 references coded [3,58% Coverage]

Yes, there have been changes. In the past cotton was doing very well, but nowadays it is not growing well, and there are now a lot of pests, that is why i am just increasing the acreage of ground nuts and maize.

Yes, there have been changes. In the past cotton was doing very well, but nowadays it is not growing well, and there are now a lot of pests, that is why i am just increasing the acreage of ground nuts and maize.

<Internals\\Transcripts\\TM 24> - § 2 references coded [1,55% Coverage]

Nowadays it’s different. I am now old, i no longer grow a large scale. I used to grow cotton on a larger scale.

Nowadays it’s different. I am now old, i no longer grow a large scale. I used to grow cotton on a larger scale.

<Internals\\Transcripts\\TM 26> - § 2 references coded [2,99% Coverage]

I decided to cut my acreage because of low returns; in the past when we grew cotton we would have good returns. With the passage of time i decided to downscale my cotton acreage.

I decided to cut my acreage because of low returns; in the past when we grew cotton we would have good returns. With the passage of time i decided to downscale my cotton acreage.

<Internals\\Transcripts\\TM 27> - § 2 references coded [1,63% Coverage]

Yes, in the past my acreage was larger than it is now. It’s mainly because nowadays i do not get enough inputs.

Yes, in the past my acreage was larger than it is now. It’s mainly because nowadays i do not get enough inputs.
